# An overview of current practice in external beam radiation oncology with consideration to potential benefits and challenges for nanotechnology

**DOI:** 10.1186/s12645-017-0027-z

**Published:** 2017-02-03

**Authors:** Raymond B. King, Stephen J. McMahon, Wendy B. Hyland, Suneil Jain, Karl T. Butterworth, Kevin M. Prise, Alan R. Hounsell, Conor K. McGarry

**Affiliations:** 1 Centre for Cancer Research and Cell Biology, Queen’s University Belfast, Belfast, BT9 7AE UK; 2 Radiotherapy Physics, Northern Ireland Cancer Centre, Belfast Health and Social Care Trust, Belfast, BT9 7AB UK; 3 Radiotherapy Physics, North West Cancer Centre, Western Health and Social Care Trust, Londonderry, BT47 6SB UK; 4 Clinical Oncology, Northern Ireland Cancer Centre, Belfast Health and Social Care Trust, Belfast, BT9 7AB UK

**Keywords:** Cancer diagnosis, Imaging contrast agents, External beam radiotherapy, Stereotactic ablative radiotherapy, Radiosensitisation, Theranostic nanoparticles, Prostate cancer

## Abstract

Over the past two decades, there has been a significant evolution in the technologies and techniques employed within the radiation oncology environment. Over the same period, extensive research into the use of nanotechnology in medicine has highlighted a range of potential benefits to its incorporation into clinical radiation oncology. This short communication describes key tools and techniques that have recently been introduced into specific stages of a patient’s radiotherapy pathway, including diagnosis, external beam treatment and subsequent follow-up. At each pathway stage, consideration is given towards how nanotechnology may be combined with clinical developments to further enhance their benefit, with some potential opportunities for future research also highlighted. Prospective challenges that may influence the introduction of nanotechnology into clinical radiotherapy are also discussed, indicating the need for close collaboration between academic and clinical staff to realise the full clinical benefit of this exciting technology.

## Background

The past two decades have seen significant advances in the technology employed in the radiation oncology environment. These advancements have enabled a move towards more individualised radiotherapy treatments, with the aim of improving their quality to obtain the optimum clinical outcome for individual patients [e.g. CHHIP clinical trial results recently published in Lancet Oncology (Wilkins et al. 2015)]. In the same period, research into the use of nanotechnology in medicine has also exploded, with a significant number of prospective clinical benefits reported. The potential medical applications of nanomaterials are vast and include tissue engineering (Walmsley et al. 2015), protein detection (Nam et al. 2003; Agasti et al. 2010) and drug and/or gene delivery (Panyama and Labhasetwara 2003; Lin et al. 2015). In addition to these applications, many studies have also proposed the inclusion of nanoparticles (NPs) into both diagnostic and radiation therapy settings, emphasising their use as potential theranostic agents (Sancey et al. 2014). The purpose of this communication is to provide an overview of the current practice in the clinical radiation oncology environment and to discuss how recent advances in this field may be combined with nanotechnology to further enhance the effectiveness of patient’s treatments. We also highlight some potential challenges that may be encountered as nanotechnology is introduced into the clinical radiation environment.

## Cancer imaging diagnostics

Patients may undergo an array of diagnostic imaging examinations as part of their oncology pathway, including x-ray computed tomography (CT), magnetic resonance imaging (MRI) and radioisotope imaging such as single-photon emission computed tomography (SPECT) and positron emission tomography (PET). Each imaging modality provides unique diagnostic data and a multimodality approach is often required to obtain the necessary information for accurate diagnosis.

### CT imaging

A common feature of x-ray-based imaging modalities is the lack of contrast between different types of soft tissue. Radiocontrast agents can be used to overcome this problem, utilising the enhanced x-ray attenuation properties of high atomic number (Z) elements (typically iodine or barium) to differentiate between tissues and accentuate additional anatomical detail, such as vascular tissue. With each evolution in CT design, there has typically also been an increase in the image acquisition rate, and scanners with gantry rotation rates of up to 4 Hz are now readily available. Combining this fast acquisition rate with contrast agents has enabled additional functional information to be acquired using CT. This technology was initially adopted in CT perfusion studies to assess blood flow to the brain in patients with suspected stroke. However, it has also been used in oncology to assess and track changes in tumour neovasculature (Perini et al. 2008), allowing clinicians to evaluate tumour response to therapeutic agents.

CT contrast agents are a highly researched application of NPs and the results of these in vitro/in vivo studies have been extensively reviewed in recent publications (Hahn et al. 2011; Shilo et al. 2012; Lee et al. 2013; Cole et al. 2015). Interest in NP contrast agents has been stimulated as a result of their flexibility, specificity and biocompatibility (Kim et al. 2010). As NPs can be formulated with a wide range of elements at their core, they can exploit high-Z elements, such as gold and platinum, which offer a desirable combination of strong absorption of x-rays and high density, providing superior contrast enhancement for lower concentrations of contrast agent. Due to their small size, NPs distribute rapidly and effectively throughout the circulatory system, enabling direct usage as vascular imaging agents or more specific targeting through conjugation to an appropriate targeting ligand. NPs also have increased clearance by the liver (Longmire et al. 2008), and therefore offer an alternative option for patients who cannot receive CT contrast due to the risk of nephrotoxicity. Finally, many in vivo studies have shown NPs to be biocompatible at concentrations which are relevant to imaging applications (Hainfeld et al. 2006).

In the oncology field, these benefits are further enhanced by NPs’ ability to penetrate the so-called ‘leaky vasculature’ formed in tumours due to the rapid proliferation of blood vessels via the enhanced permeability and retention effect (EPR). These rapidly growing vessels are permeable to nanometre-scale agents, allowing NPs to accumulate rapidly within tumour volumes, even when delivered intravenously. This may be enhanced by combining the NPs with a suitable targeting antibody or peptide to enable them to bind to the tumour cells and prolong the time spent within the tumour. In this way, tumour-specific contrast CT imaging can also be delivered, potentially offering superior tumour delineation.

A previous limitation of CT technology was its inability to discriminate between materials with similar attenuation coefficients for a single x-ray tube potential (kVp). This made it challenging to differentiate between contrast agents and physically dense tissues, such as bone (Thorsten et al. 2007). The development of dual-energy CT has gone some way to address this limitation. This technology effectively acquires two images of a patient’s anatomy using x-ray photons with either a low or high kVp spectrum. Due to the variation in attenuation coefficient with x-ray energy for different materials, the reconstructed composite images are more sensitive to the chemical composition of materials within a patient, enabling higher contrast between dense tissues and contrast agents (Johnson et al. 2007). Ongoing research into “spectral CT” detector technology may also enable similar discrimination of metal elements without the need for dual-energy acquisitions by differentiating the energy of the x-ray photons incident on its detector (Schlomka et al. 2008). The enhancement in tissue discrimination offered using these technologies could be increased further through the use of NP-based contrast agents (Cormode et al. 2010).

### MR imaging

Magnetic resonance imaging is an alternative imaging modality used regularly in oncology diagnostic examinations as it is capable of generating 3D anatomical information with superior soft tissue contrast compared to CT. The enhanced detail provided through the extensive range of advanced MRI acquisition protocols can enable clinicians to monitor the response of patients following their respective treatments. Nicolae et al. (2016) recently reviewed the application of advanced MR techniques to guide brachytherapy treatments and discussed how this enhanced guidance could be combined with NP agents to synergistically increase doses to cancerous tissue, whilst reducing the risk of radiation-induced side-effects. MRI functional diffusion maps have also been shown to provide early indications of brain tumour response to radiotherapy and chemotherapy treatments (Moffat et al. 2005). Functional diffusion maps have also been found to indicate the response of prostate bone metastasis to antiandrogen therapy (Reischauer et al. 2010).

Depending on the tissue of interest, magnetic resonance (MR) image contrast can also be further enhanced through the use of contrast agents. MRI contrast agents incorporate materials that influence local magnetic fields in their surrounding tissue, thus affecting the nuclear relaxation times used to generate MR images. Superparamagnetic iron oxide nanoparticles (SPIONs) are already in clinical use as MR contrast agents for a range of anatomical sites including the bowel, liver/spleen (Sun et al. 2008) and prostate (Coulter et al. 2015). However, the majority of MRI contrast agents are based on chelates of gadolinium. As will be discussed later, the high atomic number of gadolinium also has the potential to increase the radiosensitivity of surrounding tissues when immersed in an x-ray field and NPs that incorporate gadolinium have been proposed as theranostic agents (Sancey et al. 2014). Gadolinium chelates have also been combined with gold-based NPs creating the potential for a multimodality contrast agent that can be observed in both MR and CT images (Alric et al. 2008).

### Radioisotope imaging

Whilst CT and MRI are gaining the capability of providing functional information, they are currently unable to provide the same diagnostic value as radioisotope imaging (e.g. PET) in identifying cancerous tissue. PET studies using the Fluorodeoxyglucose (^18^F-FDG) radiotracer are commonly used in oncology investigations to identify potential cancerous tissues by highlighting regions of high metabolic activity. New radiotracers are under development and a number have been adopted clinically. One example is ligands of the prostate-specific membrane antigen (PSMA) which can be tagged with the positron-emitting ^68^Ga radioisotope (Afshar-Oromieh et al. 2013). Expression of PSMA in prostate cancer can inform clinicians of the tumour grade, pathological stage, or if it has developed castration resistance (Lütje et al. 2015). Recent in vitro studies have also demonstrated how coupling PSMA ligands to NP contrast agents can offer the facility to access this information using more readily available imaging modalities, such as CT and MRI (Wan-Chi Tse et al. 2015), whose image quality is not constrained by the limited spatial resolution inherent in PET images.

### Radiomics

It has recently been highlighted that the information contained within digital oncology images is a resource yet to be fully exploited, leading to an emerging field of research called radiomics (Lambin et al. 2012; Aerts et al. 2014). Radiomics is the conversion of digital images into mineable high-dimensional data (Gillies et al. 2016), where over 400 image features can be extracted from volumes of interest, including tumour shape, size and texture. These attributes can provide insight about a cancer’s phenotype, helping to inform the clinician of the best form of treatment. Radiomics analysis has the potential to include complementary information to other diagnostics such as genomics, pathology and blood biomarkers (Lambin et al. 2012), allowing for an improvement in the specificity of diagnostic tests. Recent radiomic studies have identified intravascular contrast-related image biomarkers that have the potential to improve the prediction of xerostomia and sticky saliva after radiotherapy with contrast (van Dijk et al. 2016). There is therefore the potential that introducing NP contrast agents into the tumour environment could also allow radiomic analysis to extract additional information from patient images regarding NP uptake and distribution within the tumour, potentially providing further insight into the cancer’s phenotype. Access to this diagnostic information should allow clinicians to further personally tailor each patient’s cancer treatment to ensure the optimum outcome.

## Radiation therapy

One application of nanotechnology that is undergoing extensive research is its incorporation into radiation therapy treatments (Wang and Tepper 2014). Results of in vivo studies have shown that radiosensitising NPs have the potential to increase the radiation dose to tumour cells, enhancing tumour control whilst sparing surrounding normal tissue and thus maximising the therapeutic ratio (Hainfeld et al. 2004). High-Z NPs, particularly gold NPs, were originally considered as radiation contrast agents due to their high atomic number. It was believed that by combining these agents with kV x-rays, the strong photoelectric absorption of x-rays by high-Z elements would increase the dose deposited in the target volume, and in turn lead to an increase in cell death. Whilst early in vivo work in this area saw successful radiosensitisation (Hainfeld et al. 2008), subsequent Monte Carlo comparisons have suggested that the increase in physical dose does not fully explain the observed radiosensitisation (Butterworth et al. 2012), which was often significantly larger than the physical dose increase.

In vitro and Monte Carlo studies have reported that the factors which impact NP radiosensitisation are yet to be fully understood (Jain et al. 2011; McMahon et al. 2011), and are therefore the subject of intensive research, but are believed to involve both physical and biological effects. Physically, in addition to increasing total absorption, the photoelectric effect typically leads to the ejection of an inner shell electron from an atom. This leaves a highly excited ion, which will proceed to release a large number of lower energy x-rays and Auger electrons. These particles deposit their energy across very short ranges, leading to highly localised damage similar to that seen in hadron therapy (Coulter et al. 2013). Such localised effects are known to drive greater biological damage than the uniform exposure delivered by x-rays, potentially contributing to their greater effectiveness. In addition, certain NP preparations have been seen to drive additional biological effects, such as mitochondrial stress or the production of reactive oxygen species, which may contribute a degree of biological sensitisation to tumour cells, in addition to their impacts on the physical dose distribution.

### Treatment planning

The application of external beam radiotherapy to treat cancer has evolved immensely in the past two decades. Increased access to volumetric images of a patient’s internal anatomy has enabled delineation of soft tissue anatomy and supported the development of more conformal treatment techniques such as Intensity Modulated Radiation Therapy (IMRT) and Volumetric Modulated Arc Therapy (VMAT). The reduction in dose to critical normal tissues afforded by IMRT has gradually led to a shift from conventional 2 Gy fractionation schedules for some treatment sites to take advantage of the radiobiological response exhibited by both tumours and surrounding normal tissue. An example of this is Stereotactic ABlative Radiotherapy (SABR) where IMRT/VMAT is used to deliver high radiation doses in a small number of treatment fractions (Chang and Timmerman 2007).

The ability to deliver radiotherapy treatments with a higher degree of conformity is also allowing clinicians to use this modality to treat diseases previously managed via other means. Oligometastatic disease [metastatic cancer which has spread to a limited number of sites (Weichselbaum and Hellman 2011)] is an example of this. Treatment of oligometastases with SABR presents an option for patients who are unsuitable for invasive surgery (Hoyer et al. 2006). The use of NP contrast agents in diagnostic tests has the potential to help identify patients with oligometastases who would benefit from this localised radiation therapy.

Despite their superior soft tissue contrast, MR images are subject to image distortion. MRI pixel values are also closely correlated with tissue proton density, which cannot be directly translated into electron densities for simulation of x-ray interaction. As a result, CT images are typically used to plan patient treatments as they are subject to less image artefact and contain accurate electron density information for modelling MV photon interactions. Unfortunately, despite the anatomical information provided by CT, delineation uncertainty is still a key source of uncertainty in radiotherapy (van Herk 2004). Whilst peer review has been highlighted as a means to reduce this uncertainty (Marks et al. 2013), lack of image contrast remains a limiting factor in determining the boundary of a cancerous volume. Contrast agents can also improve target delineation but may influence the accuracy of treatment planning algorithms (Ramm et al. 2001). However, the use of dual-energy CT may reduce the dosimetric impact of this influence (Yamada et al. 2014) and allow the benefits for NP contrast agents to be exploited further. Additionally, if NP contrast agents can be functionalised to highlight tumour burden and hypoxia regions they may offer the opportunity for a different type of conformal radiotherapy: dose painting (Ling et al. 2000). This modality introduces the concept of a “biological target volume”, a subvolume of a tumour target that can be highlighted through functional imaging modalities as having a reduced radiosensitivity. The premise of dose painting is that the treatment outcome may be improved by delivering a larger radiation dose to this subvolume. It is important to highlight that there is debate over the effectiveness of these highly conformal treatments due to other processes (such as the bystander effect), which may act to blur the biological effect that the dose distribution has on cells (McMahon et al. 2015).

The algorithms used by treatment planning systems (TPSs) to tailor treatment fields to a patient’s individual anatomy and simulate the resultant dose distribution, have evolved at a similar rate to the treatment techniques that they model. With each evolution of the algorithms, additional physical processes are incorporated into the models, improving agreement between the simulated dose distributions and the current gold standard—Monte Carlo simulations (Han et al. 2011). However, the majority of modern commercial TPSs primarily only consider Compton scattering interactions and apply density scaling to correct for inhomogeneities in a patient’s anatomy. Introducing metallic NPs into a patient’s treatment also results in the need for Monte Carlo based algorithms to accurately model the deposition of physical dose within a patient (Schuemann et al. 2016). Furthermore, for the results of these calculations to be meaningful, consistent NP uptake throughout a patient’s treatment is critical, and a stringent preparation protocol would be required.

The need for biological optimisation within treatment planning algorithms was highlighted following the introduction of IMRT (Brahme 2001) and has been incorporated into hadron therapy treatment plans to account for their increased linear energy transfer properties (Krämer and Scholz 2000). Biological optimisation takes into account the radiosensitivity of the target volume and surrounding organs at risk to generate treatment plans with the optimum therapeutic ratio. Similar optimisation would be essential when employing NP radiosensitisation and would require accurate characterisation of NP interaction with the x-ray spectrum of the irradiation field as a function of nanoparticle material, size and concentration (Schuemann et al. 2016).

### Treatment delivery

The introduction of highly conformal techniques has increased the need for pre-treatment verification imaging to confirm accurate positioning of the patient. As a result, image-guided radiotherapy (IGRT) has developed at a similar pace to IMRT techniques. Most manufacturers now offer linacs with kV-based on-board imagers, capable of generating high-contrast planar images, as well as volumetric Cone Beam CT (CBCT) images of the patient’s internal anatomy at the time of treatment. Whilst CBCT images provide invaluable volumetric information, the acquisition method has a number of image quality limitations compared to their diagnostic CT counterparts (Srinivasan et al. 2014). Studies using IGRT with contrast-enhanced CBCTs have reported an improvement in target localisation accuracy of liver tumours in patients who have undergone chemoembolization using iodine-based Lipiodol contrast agent (Yue et al. 2012). However, further consideration needs to be given concerning administering contrast agents (including NPs) during treatment, including the implementation of strict administration protocols to enable consistent contrast distribution and accurate dose simulation.

The volumetric information contained within CBCT images can also be of additional benefit after a patient’s treatment to determine the dose the patient actually received (Hatton et al. 2011). The range of IGRT solutions currently available (such as CBCT, planar imaging and fluoroscopy systems) may also allow for the evaluation of changes in NP uptake and provide a means to monitor patient response to their treatment. With the rapid adoption of IGRT, there is discussion about incorporating the imaging dose into a patient’s radiotherapy prescription (Hyland et al. 2014; Alaei and Spezi 2015). If NPs were also to be introduced into the clinical environment then the imaging dose may become more clinically significant through their inherent kV dose-enhancement properties (Butterworth et al. 2012).

As mentioned previously, the main benefit to using NPs during treatment is the potential dose enhancement that results from the interaction of the MV treatment field with the NPs. With targeted uptake of NPs, there is the potential to increase the dose to the tumour whilst maintaining or reducing dose to surrounding normal tissue. This is particularly relevant to SABR treatments, where a single geometric miss in the hypofractionated treatment schedule can result in a reduction in tumour control and/or an increase in toxicity effects. There is also a trend towards the increased use of flattening filter free (FFF) fields to deliver SABR treatments due to their significantly higher dose rates. Whilst previous concerns regarding synergistic biological effects with these higher instantaneous dose rates have so far proved to be unfounded (King et al. 2013), in vitro studies have indicated that NP interaction with the softer photon spectrum associated with FFF fields may enhance their dose deposition properties further (Detappe et al. 2016). Further study is therefore required to fully evaluate the possible physical and biological enhancements that can result from combining FFF fields with NPs. An intriguing potential alternative application of nanoparticles in radiotherapy is as vectors for radiation protection agents (Schweitzer et al. 2010). An in vivo study with mice demonstrated that intravenous delivery of melanin-coated NPs reduced bone marrow toxicity during external beam radiotherapy without affecting tumour control (Schweitzer et al. 2010).

### Emerging technologies

In light of the benefits that IGRT has brought to radiotherapy treatments, manufacturers are investigating methods to provide additional imaging information at the time of treatment. To take advantage of the superior soft tissue contrast, MRI devices have been combined with Cobalt sources and MRI-guided linac systems are also in development (Oelfke 2015). A PET-based linac system is also under development with the premise that the tumour target essentially guides the treatment delivery (Fan et al. 2012; Yang et al. 2014). These combined treatment/imaging modalities provide additional avenues of research to determine how the diagnostic and therapeutic merits of nanotechnology can be used to further enhance their therapeutic benefit. Such studies are of importance as little is known about the potential dosimetric effects of introducing NPs into overlapping magnetic and x-ray fields.

The number of radiotherapy centres using beams of protons or heavy ions (hadrons) to treat tumours is rapidly increasing, with approximately 70 clinically active centres worldwide and another 50 centres being built or planned (PTCOG 2016a, b). The dose deposition characteristics of hadrons make them a very attractive choice of radiation therapy. Hadrons have a low entrance dose and deposit the majority of their dose in a well-defined Bragg peak, determined by particle energy, with rapid dose fall-off subsequent to this. This generates dose distributions with superior conformity around the target volume compared to photons, which is particularly important with tumours that are directly adjacent to a radiosensitive organ. Hadrons also cause highly localised damage on the sub-cellular scale resulting in enhanced radiobiological effectiveness. Monte Carlo simulations have indicated that metal NPs can also provide dose enhancement during hadron therapy (Lin et al. 2014). This enhancement has been confirmed in in vitro studies using He^2+^ or C^6+^ beams combined with gadolinium-based NPs (Porcel et al. 2014), where it was observed that the dose enhancement stemmed from the activation of early nano- and sub-nanosized processes in the cytoplasm, far from the nucleus.

Radiation therapy is also commonly combined with other well-established treatment modalities (such as chemotherapy) to enhance treatment outcome (Harrington et al. 2011). The use of NPs to deliver chemotherapeutic agents has been extensively reviewed (Brigger et al. 2002; Wang et al. 2012). As with their application in radiotherapy, one of the main advantages to employing NPs as chemotherapy vectors is the improved targeting potential. Clinical translation of this localisation attribute has been investigated in clinical chemotherapy studies using a number of chemotherapy agents which were recently reviewed by Wang and Tepper (2014). There is also the potential to use NPs as chemoradiotherapy enhancing agents for concurrent therapies, where increased uptake in the tumour can enhance tumour control whilst reducing unintentional side-effects caused by damage to normal tissues (Wang and Tepper 2014). NPs can also be employed to build upon the success of recent radionuclide therapies, such as clinical trials that employed radium-223 (Hoskin et al. 2014). With radioisotope-tagged functionalised NPs, there is the potential to translate this success to other metastatic diseases (Barreto et al. 2011).

## Limitations and challenges

As highlighted throughout the text, there are a number of challenges that must be overcome before nanotechnology can be introduced into routine clinical practice in radiation oncology departments. Chief amongst these is the development of clinical trials to evaluate the efficacy of NP-mediated diagnosis and/or treatment and to quantify the clinical benefit to patients. However, before clinical trials can be formulated a number of other limitations must be addressed.

To be clinically applicable, NPs must fulfil a very stringent set of criteria. These include biocompatibility, to reduce the risk of treatment side-effects; desirable pharmacokinetic properties, providing both high target specificity and good dispersion throughout the target volume; and efficacy in driving the desired effect in target cells (whether imaging- or therapy-related). In vitro and in vivo studies of these properties have shown that they are not easy to predict from individual particle characteristics. Rather, they are the result of a complex interplay between particle size, shape, material and coating, amongst other factors. Because of this interplay, piecewise optimisation of NP properties is not likely to be feasible, but particles must be subjected to comprehensive preclinical studies in vitro and in vivo considering all of these effects before being translated into clinical studies (Shuemann et al. 2016).

There also remains the significant challenge of manufacturing NPs on a practical scale. Most studies make use of either in-house NP preparations, or commercial particles produced on a scale suitable for laboratory experiments. Many of these preparations are often expensive—with bare unconjugated nanoparticles costing on the order of $10,000/gram of gold, and bespoke functionalised particles having significantly greater effective costs. The effective scalability of novel nanoparticles therefore requires consideration when developing new approaches. However, demonstration of clinical proof of principal should increase interest and competition from large pharmacological companies.

Finally, effective modelling of the physics of NP interactions remains a pressing challenge. The introduction of high-Z materials into tissue is currently poorly incorporated by most TPS, and more robust, validated physics models are needed to ensure that the quality of radiotherapy plans are not reduced through the introduction of nanoparticles as part of treatment planning.

## Conclusions

New technologies are rapidly being introduced into the radiation oncology environment with many technologies offering features that are yet to be fully exploited. In the emerging era of increased personalisation of oncology treatments, nanoparticles can provide an extremely useful tool in every stage of a patient’s radiotherapy experience from diagnosis, to treatment and subsequent follow-up monitoring. As this technology begins to be introduced into the clinical environment, close collaboration between academic and clinical staff is essential to identify potential challenges and opportunities and ensure that this promising technology provides the maximum benefit to patients.

## Abbreviations

CHHIP: conventional or hypofractionated high dose intensity modulated radiotherapy for prostate cancer
CBCT: cone beam computed tomography
CT: computed tomography
EPR: enhanced permeability and retention effect
FDG: Fluorodeoxyglucose
FFF: flattening filter free
IGRT: image-guided radiotherapy
IMRT: intensity modulated radiation therapy
MR: magnetic resonance
MRI: magnetic resonance imaging
NP: nanoparticle
PET: positron emission tomography
PSMA: prostate-specific membrane antigen
PTCOG: Particle Therapy Co-Operative Group
SABR: stereotactic ablative radiotherapy
SPECT: single-photon emission computed tomography
SPIONs: superparamagnetic iron oxide nanoparticles
TPS: treatment planning system
VMAT: volumetric modulated arc therapy
